# The Safety–Autonomy Grid: A Flexible Framework for Navigating Protection and Independence for Older Adults

**DOI:** 10.1093/geront/gnaf111

**Published:** 2025-03-17

**Authors:** Kelly Marnfeldt, Kate Wilber

**Affiliations:** Leonard Davis School of Gerontology, University of Southern California, Los Angeles, California, USA; Leonard Davis School of Gerontology, University of Southern California, Los Angeles, California, USA

**Keywords:** Ethics in care, Independence and self-determination, Person-centered decision-making, Quality of life, Supported risk enablement

## Abstract

The balance between safety and autonomy becomes a central tension for older adults with cognitive impairments, complex health needs, or age-related vulnerabilities. This tension arises across individual decision-making, interpersonal relationships, in institutional environments, and in broader societal structures. The Safety–Autonomy Grid provides a practical framework to address this evolving challenge, helping individuals such as family members, caregivers, and policymakers understand this tension and better navigate the balance between safety and independence for older adults. This framework promotes more balanced and reflective decision-making to promote person-centered outcomes that preserve dignity and independence while ensuring appropriate protection. The framework also highlights the pragmatic balance between safety and autonomy as a fluid, evolving process that can be applied across caregiving, legal, and policy contexts. While the framework presents numerous applications, its adoption may face certain challenges, such as institutional resistance, cultural biases, and long-established protocols that make it difficult to embrace new approaches. Ultimately, the Safety–Autonomy Grid serves as a practical tool for promoting more equitable decision-making, ensuring that older adults’ rights are preserved and that decisions are made with careful consideration, free from bias or undue haste.

The balance between safety and autonomy becomes a central tension as people encounter age-related changes, particularly when cognitive impairment or complex health needs arise (Dickins et al., 2018). This tension can occur at an individual decision-making level, within interpersonal relationships, in institutional environments, or in broader societal structures (Higgs & Gilleard, 2016). When safety is prioritized at the expense of autonomy, protective measures can unintentionally diminish a person’s quality of life by overriding their preferences and eroding their sense of agency (Berry et al., 2015; Moyle et al., 2011).

This dichotomy between protecting adults from harm and respecting their right to self-determination can become more pronounced at later stages of the life course and has shaped many policies and interventions (Baltes & Silverberg, 2019). However, the assumption that safety and autonomy exist as a binary choice has been challenged by older adults and their advocates in favor of a more nuanced approach, acknowledging that the ability to make and act on one’s own decisions fluctuates greatly across the life course (Ekelund et al., 2014).

This approach emphasizes that vulnerability is shaped more by health and personal circumstances than by age. As Martinez et al. (2023, p. 980) argue, “Autonomy versus safety is, to some degree, a false dichotomy,” suggesting that these two forces can coexist within a thoughtfully designed care system. This perspective acknowledges that safety and autonomy, though often seen as opposing, are interdependent aspects of risk management.

Population aging intensifies the need to address this issue more deliberately and equitably. Many professionals and caregivers prioritize protection over independence due to risk aversion, concerns about liability or litigation, and underlying biases, including ageist attitudes (Hoe et al., 2023; Wright, 2010). Ageist assumptions often lead to an overemphasis on safety at the expense of autonomy, as decision-makers may presume older adults are inherently fragile or incapable of managing risk. Yet, research demonstrates that even older adults with dementia living in residential care benefit from being included in decisions. Seeking their input and respecting their preferences has been shown to improve self-esteem and quality of life (Daly et al., 2018).

Longer life expectancy can be associated with increasing rates of health conditions, including chronic illnesses, cognitive changes, and disabilities, which may contribute to heightened risks in later life (Medina et al., 2020). These shifts in health and functional status place safety and autonomy to the forefront of care decisions for older adults. Multimorbidity—managing multiple chronic illnesses—compounds these challenges, as the vast majority of older people wish to age in place, even as their needs increase dramatically (AARP, 2021). Without clear frameworks to guide decisions, protective measures driven by conventional wisdom, advocacy priorities, and concerns about potential risks such as falls, cognitive challenges, or financial exploitation can unnecessarily restrict independence, prioritizing safety at the expense of autonomy.

The tension between “doing too much”—imposing overly restrictive care—and “doing too little,” which may expose individuals to preventable risks, is a central challenge for caregivers and policymakers (Martinez et al., 2022). Measures such as full guardianship, or restricting movement out of fear of falls, can lead to premature or unnecessary institutionalization, loss of dignity, and poor mental health, due to depression, anxiety, and diminished self-worth (Wright, 2010). Conversely, unsupported independence, such as unsupervised living with cognitive decline, poses serious health and safety risks (Long et al., 2015). While the need for support can increase vulnerability to abuse across all ages, older adults may face heightened risks due to factors associated with aging, such as physical frailty, social isolation, or increased reliance on caregivers, which can be exacerbated by aging-related challenges and societal barriers. Even older adults without cognitive impairment who require support to maintain independence may be at increased risk of elder abuse and neglect when needed support is absent (Burnes et al., 2015).

Revoking an older adult’s civil liberties through legal means may be necessary when cognitive decline or other vulnerabilities prevent self-protection. While measures like full guardianship are intended to prevent harm, they can sometimes lead to abuse, causing severe emotional, psychological, and financial suffering (Anetzberger, 2005; Wood, 2012). Such arrangements may trap individuals in untenable and difficult-to-reverse legal predicaments where guardians, whether through negligence, misunderstanding, or disregard for rights, compromise their dignity and well-being (Kohn, 2018; Miller, 2018).

Despite the tension between safety and autonomy, current thinking often oversimplifies these concepts. Safety is frequently interpreted as protection from harm at all costs, while autonomy is viewed as unrestricted freedom. This binary framing fails to capture the complexities of elder care and decision-making, failing to account for the gray areas in between (Strech et al., 2013). A more nuanced approach recognizes safety and autonomy as a spectrum, requiring carefully calibrated trade-offs tailored to individual needs and circumstances.

What is needed is a framework that balances safety and autonomy by addressing the harmful extremes often seen in elder care. On one end, overly restrictive measures, such as premature institutionalization, erode dignity, and independence (Wright, 2010), while at the other, insufficient support leaves individuals exposed to preventable harm (Li et al., 2023). As these examples demonstrate, such tensions aren’t hypothetical; they can turn into real-world challenges that strain individuals, caregivers, healthcare providers, and legal systems.

By rethinking safety and autonomy as interdependent rather than opposing, we can better navigate the complexities of care, alleviate systemic pressures, and uphold the rights and dignity of older adults. To that end, this paper introduces the Safety–Autonomy Grid, a structured framework to help family members, healthcare providers, legal systems, and older adults navigate these tensions more deliberately. Understanding how safety and autonomy operate across different levels of decision-making—from individuals to families to care systems—is essential for applying the Grid in practice.

## The Ecosystem of Safety and Autonomy in Aging

While adults of any age may experience limitations in independence due to illness or accident, discussions about restricting personal freedoms are rarely applied to younger, nondisabled individuals. When discussing older adults, however, these conversations often take on a protective and, at times, paternalistic tone as family members, caregivers, and healthcare providers focus on minimizing risks. This shift reflects how decision-making processes can change over time, especially when individuals face increasing challenges or evolving needs. The question shifts from “How do I recover and return to my usual routines?” to “What does independence look like for me long term, and how can I maintain it as challenges arise?”

**Figure 1.**
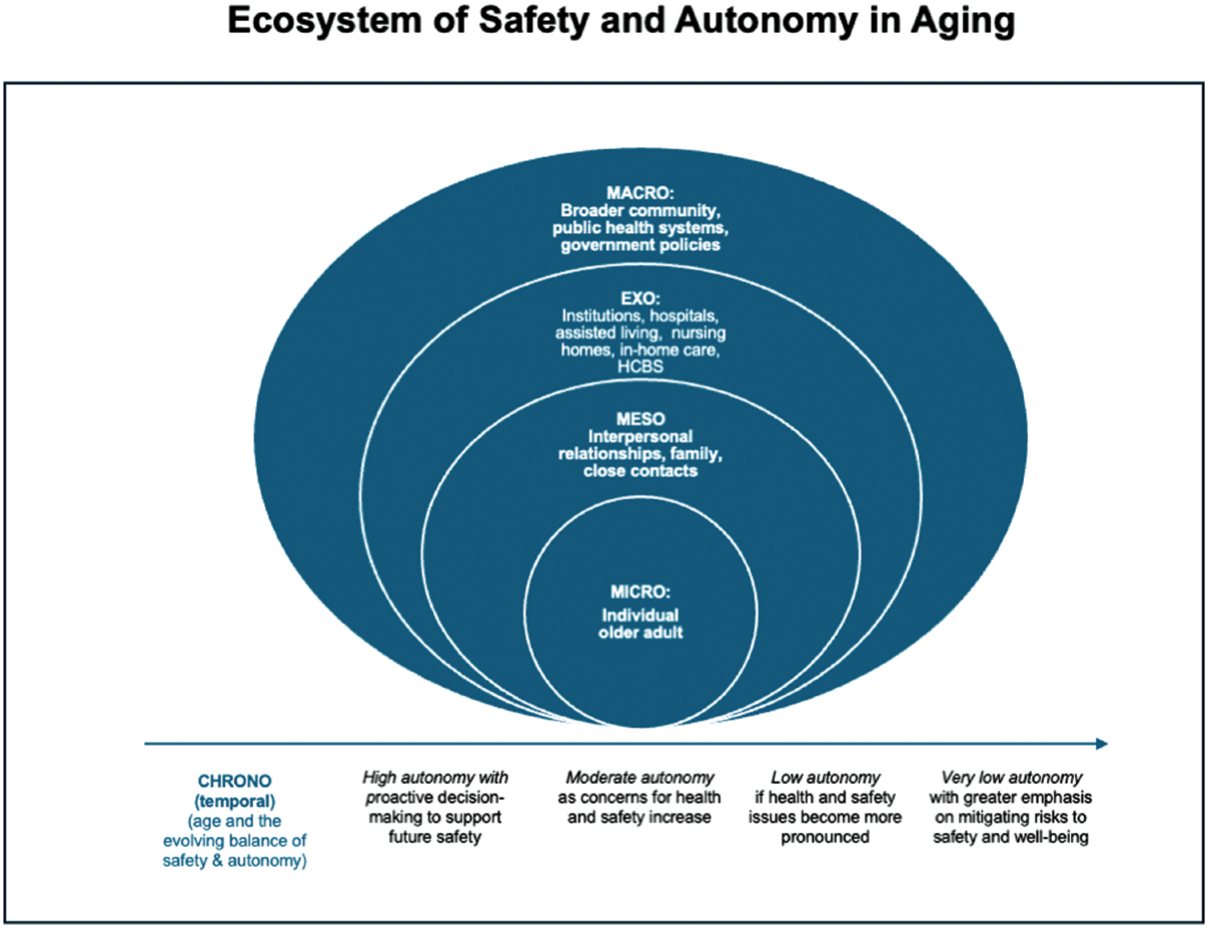
Ecosystem of safety and autonomy in aging. This figure illustrates the multidimensional framework of Bronfenbrenner’s Social Ecological Model (1979), showcasing how decision-making regarding autonomy and safety for older adults occurs across various levels of influence. The model spans four interconnected contexts: personal (individual-level factors); relational (family, caregivers, and close networks); institutional (healthcare systems, long-term care facilities, legal entities); and societal (social norms, policies, and cultural attitudes). The temporal (chrono) dimension reflects a general shift from higher to lower autonomy over time, though this progression is not strictly linear. Older adults may move between levels of autonomy based on changes in health, caregiving, social environments, or personal circumstances.

The ecosystem of decision-making in aging is vast, spanning personal, relational, institutional, and societal contexts. Bronfenbrenner’s Social Ecological Model (1979) provides a multidimensional framework for understanding how various layers of influence—from personal relationships to social policies—intersect and shape the decisions surrounding autonomy and safety for older adults. By also considering how these systems interact over time, we can gain deeper insights into the complexities of decision-making processes for aging individuals (see Figure 1).

At each level, decisions balancing autonomy and protection take place in both formal and informal contexts. Family choices, institutional policies, and legal structures intersect to shape an individual’s ability to retain independence. Even well-intentioned decisions must balance mitigating demonstrable risks with preserving independence, recognizing that this negotiation evolves across contexts and over time.

### Individual, Micro Level

At the individual level, an older adult’s desires, needs, and capacities come into direct focus. Decisions about daily routines, health management, or long-term care involve navigating priorities and preferences amid changing abilities and challenges. When circumstances change or vulnerabilities increase, individuals may face choices about driving, medication management, or living independently. Many older adults maintain independence and agency by making proactive adjustments, such as limiting driving to daylight hours rather than giving it up entirely. These adaptations demonstrate how autonomy can be preserved through thoughtful risk management (Carstensen et al., 2003).

### Interpersonal, Meso Level

At the interpersonal level, family members, caregivers, and close contacts play a central role in balancing safety and autonomy. As health and support needs increase, family dynamics often shift, and the roles of adult children, spouses, or friends may expand to include caregiving tasks. Decisions about an older adult’s independence are often influenced by those closest to them. However, the informal nature of caregiving can complicate roles, with some family members unintentionally overreaching due to protective instincts, while others may struggle to provide adequate support.

### Institutional, Exo level

At the institutional level, the ecosystem includes healthcare providers, legal professionals, social workers, and others in formal settings. Safety concerns often compete with individual autonomy in hospitals, assisted living facilities, and nursing homes, especially in high-risk situations. For instance, hospital discharge planning decisions—such as home care, rehabilitation, or long-term care—are shaped by safety needs and the individual’s ability to live independently. However, institutional considerations like costs and liability frequently shape these priorities. Legal systems also play a pivotal role, particularly in guardianship cases, where autonomy may be significantly curtailed to prioritize protection from harm.

### Community and Policy, Macro Level

At the community and policy level, public health systems, government policies, and social services establish the frameworks for debates on safety and autonomy. Home and community-based services, for example, aim to support older adults to remain at home, reflecting societal values of independence and dignity. However, disparities in access to these services—driven by structural barriers in funding, availability, and eligibility—leave some older adults without adequate care, forcing difficult compromises when meaningful choices are unavailable. These broader societal structures critically influence how safety and autonomy are balanced, with significant implications for older adults’ quality of life.

### Temporal, Chrono Level

Finally, at the chrono level, the balance between autonomy and safety evolves over time, shaped by changes in mobility, cognition, and health. While individuals may begin their older years with very high levels of independence, emerging risks and changes often require ongoing reassessment by caregivers, healthcare providers, and the individuals themselves.

The Safety–Autonomy Grid was created to address this evolving tension by offering a framework to understand how various actors—from family members to policymakers—navigate these considerations. Rather than framing safety and autonomy as opposing forces, the grid conceptualizes them as a spectrum of trade-offs, promoting personalized, adaptive approaches that respect dignity, independence, and changing needs throughout the life course.

### Theoretical Framework

Inspired by the Blake Mouton Managerial Grid (Blake & Mouton, 1961), the Safety–Autonomy grid shifts the focus from an “either-or” to “both-and” proposition, balancing concern for safety and autonomy in decision-making processes.

Originally developed to assess leadership styles in organizational management, the Managerial Grid is used to evaluate how leaders balance concern for people with concern for production within business contexts (Blake & Mouton, 1961). This conceptual precedent—balancing two equally important but sometimes competing priorities—made it a natural foundation for adapting the grid to the ethical tensions between safety and autonomy in aging-related decisions.

In this adaptation, the Safety–Autonomy Grid serves as a practical tool to guide decision-makers—at the individual, interpersonal, institutional, and societal levels—to assess where their priorities fall on the spectrum and to ensure that preserving autonomy is given equal weight to protecting from harm.

**Figure 2.**
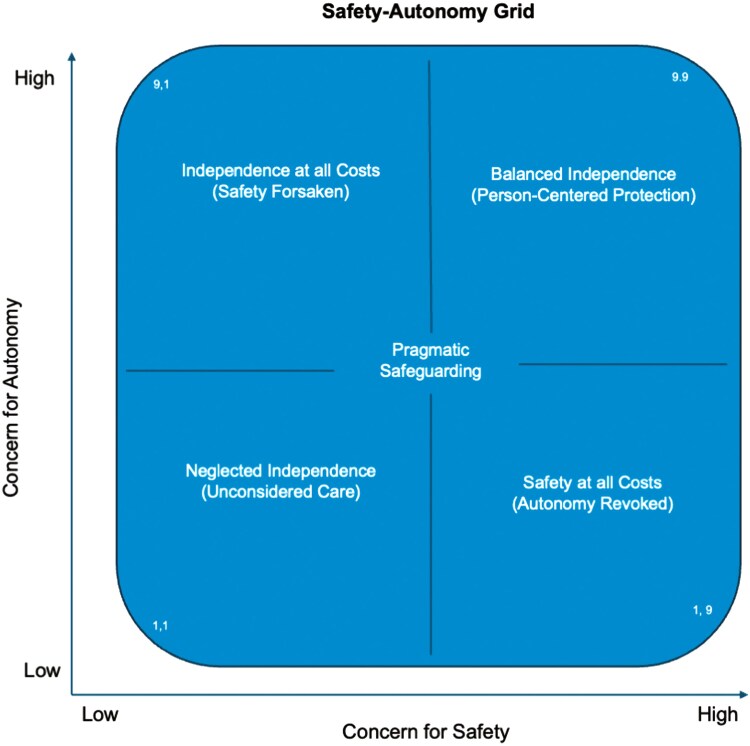
Safety–Autonomy Grid. This figure presents the Safety–Autonomy Grid, a reflective framework for evaluating the balance between safety and autonomy in decision-making for older adults. The *X*-axis represents concern for safety, which includes harm prevention across physical, emotional, and financial domains. The *Y*-axis reflects concern for autonomy, emphasizing freedom, independence, and control. The grid is divided into four quadrants, visually mapping the tension between these priorities. Stakeholders are encouraged to assess where their decisions fall within this grid to identify blind spots and reflect on how their actions may unintentionally prioritize safety at the expense of autonomy. The framework highlights the importance of balancing protection with autonomy to preserve older adults’ civil liberties and dignity.

In the Safety–Autonomy Grid, the *X*-axis reflects concern for safety, emphasizing harm prevention across physical, emotional, and financial domains, while the *Y*-axis represents concern for autonomy, highlighting control, freedom, and independence. This framework serves as a reflective tool encouraging stakeholders to visualize the tension between these priorities and recognize where their actions may unintentionally prioritize safety at the expense of independence. By assessing where their decisions fall on the spectrum, decision-makers are encouraged to recognize potential blind spots, including how their choices may unintentionally restrict an older adult’s autonomy. The framework also prompts reflection on the profound consequences of such decisions, particularly when civil liberties are at stake, and encourages decision-makers to examine their own tolerance for risk and to explore alternatives that preserve agency while addressing safety concerns (see Figure 2).

The Safety–Autonomy Grid aligns with and expands upon existing care approaches that emphasize dignity, autonomy, and safety. Person-centered care models are widely discussed in gerontology and healthcare, emphasizing the importance of respecting an older adult’s choices while ensuring their safety (American Geriatrics Society Expert Panel on Person‐Centered Care, 2016). Similarly, shared decision-making frameworks prioritize involving older adults in care decisions, even when safety is a concern (Pel-Littel et al., 2021). The Safety–Autonomy Grid builds on these principles, offering a visual framework that explicitly maps the tension between these two priorities, encouraging more nuanced and individualized decision-making.

While the Safety–Autonomy Grid builds on principles of person-centered care and shared decision-making, it also shares structural similarities with other models. For example, the Social Discipline Window (McCold & Wachtel, 2001) balances control (discipline) and support (encouragement) within restorative justice contexts. Unlike these frameworks, however, the Safety–Autonomy Grid is specifically designed to address the ethical complexities of decision-making with and for older adults and individuals with disabilities, where trade-offs between safety and autonomy have a direct and lasting impact on independence and well-being.

Ultimately, the Safety–Autonomy Grid offers a structured, reflective tool for addressing the complex balance between safety and autonomy across diverse care and decision-making contexts. By visually mapping these priorities, the grid equips stakeholders to consciously reassess their approaches and align decisions more closely with an individual’s needs, preferences, and values. In doing so, it fosters decision-making that prioritizes well-being, dignity, and quality of life—critical considerations in situations where such choices have lasting impacts on independence and self-determination.

### The Safety–Autonomy Quadrants in Practice

To better illustrate the range of possible outcomes in caregiving decisions, we will begin with the least desired outcome, Quadrant III, “Neglected Independence (Unconsidered care),” and progressively move toward the optimal balance represented by Quadrant I, “Balanced Independence (Person-Centered Protection).” The case examples presented below are hypothetical and are intended to illustrate the practical applications of the Safety–Autonomy Grid across various individual, caregiving, institutional, and societal contexts.

### Quadrant III—Neglected Independence (Unconsidered Care)

In this quadrant, neither safety nor autonomy is prioritized. It represents scenarios where older adults are left to fend for themselves without adequate support, including situations of self-neglect and social isolation. This level of disregard for both safety and autonomy can occur in family caregiving settings, within institutional environments, and society at large. In some situations, older adults may desire assistance but are unable to access it due to isolation or limited caregiver capacity. Additionally, self-neglect may stem from a lack of insight or fear that seeking help could lead to pressured safety interventions, such as placement in a care facility.

The starkest societal example can be seen among unhoused older adults, where the social safety net has entirely collapsed. At the institutional level, an older person who lacks sufficient support might be discharged with inadequate home care services in place. Unconsidered care can also happen at the individual level, where an older adult may have a family member present, but that person may lack the mental, emotional, or physical capacity to provide adequate care. In some cases, an older adult may not fully recognize their own unmet needs, and a tool like the Safety–Autonomy Grid could help them reflect on their situation, identify risks, and initiate conversations with family members or care providers before a crisis occurs.

This quadrant illustrates the dangers of passive neglect, where lack of care leads to preventable harm. Communities, institutions, and families who fall into this quadrant must ask: “Have we unintentionally left the person vulnerable due to a lack of oversight or resources, or are there deeper systemic issues at play?”

### Quadrant IV—Safety at All Costs (Autonomy Revoked)

This quadrant represents an overemphasis on safety, often resulting in the complete loss of autonomy. Common examples include full guardianship, restrictive or premature institutional care, or caregiving that limits personal decision-making. This overemphasis can occur at both individual and institutional levels, leading to significant restrictions on personal choice and independence.

In certain situations, older adults at the center of these conversations may recognize this overprotective dynamic developing and could use the Safety–Autonomy Grid as a framework to communicate their preferences before external decisions override their autonomy. For older couples aging together, the grid could also support joint discussions, helping partners articulate how they each balance their own safety and independence, especially if one partner’s health or cognitive needs change faster than the other’s.

However, when these proactive conversations do not occur—or when external decision-makers disregard older adults’ expressed wishes—overprotection often unfolds.

For example, consider an otherwise healthy older woman living alone who one day suffers a fall in her home. Her adult children in another state are alarmed and worried and exert undue pressure on her to go into an assisted living facility against her wishes. Or, at the institutional level, consider an older adult admitted to a rehabilitation facility who lives alone with no nearby relatives. If concerns are raised about their cognitive abilities—sometimes without thorough assessment—the facility may petition the court for guardianship to ensure authorized decision-making throughout their stay and discharge planning. Full guardianship is frequently imposed without considering less restrictive alternatives, even when individuals retain certain capacities. This can lead to the denial of financial, medical, and personal autonomy, even when appropriate support is available.

This quadrant raises significant concerns about overprotection and the potential for elder abuse in the guise of paternalistic safeguarding. Decision-makers—whether probate judges, family members, or institutions—must ask themselves: “Are we unnecessarily stripping away autonomy in the name of safety?” or “Have we meaningfully explored community-based supports or less restrictive alternatives?” The ethical questions raised here underscore the importance of carefully weighing immediate protective benefits against the long-term consequences of removing decision-making power from older adults.

### Quadrant II—Independence at All Costs (Safety Forsaken)

This quadrant prioritizes autonomy above all else, even when significant risks are present. Consider an older adult with moderate cognitive impairment who insists on living independently but refuses needed help. Their fierce commitment to independence can create deep emotional stress for family members and caregivers. Family history and dynamics can also make these situations undeniably challenging. Perhaps this individual has always been strong-willed or perhaps promises were made that they could remain in their own home, making it difficult to challenge their choices.

In these cases, the Safety–Autonomy Grid could again serve as a self-reflection tool for the older adult themselves, helping them examine whether their desire for autonomy aligns with their actual capacity and risks. Using the grid in this way could encourage proactive conversations between the older adult and their family or care team, reducing the likelihood of escalating conflict or emergency interventions.

This quadrant underscores ethical dilemmas for caregivers and decision-makers who may struggle between honoring an older adult’s choices while also protecting them from harm. Caregivers might ask: When does preserving independence verge on neglect? Are we prioritizing dignity, or are we avoiding difficult conversations about necessary care? How can we address safety concerns without disempowering the individual? These questions reveal the ongoing tension between responsibility and autonomy, especially in cases of cognitive decline or physical frailty.

### Quadrant I—Balanced Independence (Person-Centered Protection)

This quadrant represents the ideal of modern eldercare models, where safety and autonomy coexist within a person-centered care approach. Here, conditions need to be created where caregivers have access and knowledge to be able to take advantage of tools such as adaptive technologies, individualized care plans, and collaborative decision-making, ensuring that older adults can maintain as much independence as possible while minimizing risks.

For example, a person with early-stage dementia might continue to live at home with the support of assistive technologies, such as location trackers, medication reminders, and fall detectors. They may also receive regular home visits from home care workers who assist them with daily tasks while promoting independence. Family members and healthcare providers would ideally work closely to adjust the care plan as the person’s condition evolves.

In addition to input from family members and care providers, older adults can independently and proactively engage with the Safety–Autonomy Grid in this quadrant, using it as a tool to articulate their preferences, name the risks they are (and are not) willing to accept, and actively participate in shaping their care plans.

Decision-makers in this quadrant are encouraged to ask: How can autonomy be preserved while minimizing risks? or What decisions can the older adult continue to make independently, and where is additional support needed? This approach adheres to the guiding principles of person-centered care, where the individual’s preferences are central, even as they receive protection from harm.

### Pragmatic Safeguarding: Navigating the Spectrum

The middle section of the grid reflects that there is a pragmatic balance between safety and autonomy and acknowledges it is a fluid, evolving process, rather than a fixed, rigid state. For many individuals and families, resources may be limited, and complete safety may not be achievable. For example, a family caring for an older adult with mobility challenges installs grab bars and a stairlift to ensure safety, while supporting their independence in daily routines and activities. They regularly assess the individual’s capacity and adapt care as needs change. Rather than prioritizing maximum protection—which could lead to restrictive interventions—the family takes an adaptable approach, accepting manageable risks rather than aiming to eradicate all potential hazards.

Individuals in this quadrant must ask: How do we maintain a dynamic balance between independence and safeguarding as needs shift? This quadrant is the most realistic for many caregivers and decision-makers, as it also acknowledges that resources and care needs vary greatly. Unlike the aspirational aim of Quadrant I, Pragmatic Safeguarding reflects the reality that perfect solutions rarely exist. Here, safety and autonomy are navigated through small, adaptive steps and compromises that prioritize progress over perfection.

### Applications and Real-World Implications

This process of balancing safety and autonomy does not occur in isolation; rather, it unfolds within a broader web of relationships, institutions, and policies that influence decision-making. The Safety–Autonomy Grid draws on Bronfenbrenner’s Social Ecological Model, which highlights how safety–autonomy tensions are shaped not only by individual choices but also by the broader ecosystem of family, healthcare, legal systems, and policy environments.

The Safety–Autonomy Grid is a practical decision-making tool designed to help a wide range of stakeholders evaluate and reflect on how their decisions balance the competing priorities of safety and autonomy for older adults. The grid encourages both older adults and those involved in their care to visualize where their actions fall on the spectrum between protecting the individual from harm and allowing them the freedom to make their own choices.

Recent research highlights that older people experience autonomy not as a fixed state, but as something shaped by personal values and evolving purpose over time—something often missed in static, “snapshot” assessments (Taylor et al., 2024). The Safety–Autonomy Grid directly addresses this gap by offering a flexible tool that supports value-driven decision-making, helping older people and those around them revisit and realign decisions as circumstances and personal meaning shift. In addition, the grid reinforces individual agency by prompting older adults to articulate their own preferences whenever possible, while also encouraging those around them to remain attentive to these preferences even as health and cognitive abilities change over time.

Broader evidence underscores the importance of tools that counteract common tendencies toward paternalism and overprotection. Chen and Zhang (2024) found that well-intentioned paternalistic help can actively undermine autonomy, contributing to poorer health outcomes and reduced survival. Similarly, the COVID-19 pandemic has exposed deep ethical tensions in long-term care, highlighting the risk that future public health crises could repeat these patterns (Davis et al., 2021). These patterns highlight why frameworks like the Safety–Autonomy Grid are necessary—to help decision-makers remain mindful of these risks and actively work to balance safety and autonomy in ways that honor both protection and dignity.

The grid further encourages self-awareness and reflection, providing a visual tool to assess whether values and actions align with an older adult’s best interests or skew too much toward either end of the spectrum, potentially compromising one for the other. For instance, a family caregiver who prioritizes independence may realize they are not providing enough safety measures, or a healthcare professional might recognize that they are thinking about a person’s capacities too narrowly and unnecessarily limiting their autonomy. By identifying these tendencies, individuals have an opportunity to stop and think and perhaps adjust their approach to better reflect the older adult’s needs and preferences.

The grid offers decision-makers a practical tool to move beyond instinct, habit, and ingrained cultural or systemic biases, including ageism. By challenging these assumptions, it fosters thoughtful, evidence-based decisions that respect the complexities of aging and individual variation. In situations where the balance between safety and autonomy is unclear, the grid provides a shared reference point for navigating the gray areas, prompting meaningful conversations and encouraging more equitable, person-centered care. Ultimately, the grid aims to disrupt entrenched biases, helping stakeholders achieve a balance that honors both dignity and protection.

## Barriers and Limitations to Adoption

While the Safety–Autonomy Grid offers a structured approach to engage in a dialogue with oneself, with an older adult, and within systems of care, its practical application may present several barriers and limitations. Recognizing and addressing these barriers is essential to ensure the grid’s potential as a transformative tool for decision-making in elder care. Below are some of the key barriers that must be addressed for the grid to be effectively implemented:

### Institutional Resistance

The grid marks a departure from the safety-first status quo. Courts, healthcare systems, and care facilities may resist adopting new frameworks, especially in populations perceived as vulnerable. This resistance can slow its’ implementation, as institutions may prioritize risk aversion, driven by concerns over reputational harm, potential litigation, and the desire to minimize adverse events. Additionally, deeply ingrained practices within rigid, bureaucratic systems, further complicate implementation. Integrating the Safety–Autonomy Grid into existing processes, would require not only structural adjustments but also cultural shifts—challenging longstanding preferences for safety and efficiency over more nuanced, person-centered approaches.

### Lack of Familiarity and Training

For the Safety–Autonomy Grid to be effectively adopted, stakeholders across diverse domains—physicians, judges, legal professionals, caregivers, and others—must first understand its principles and practical applications. Without sufficient exposure or adequate training, these individuals may fail to see its value or struggle to apply it meaningfully in real-world settings. This learning curve presents a significant barrier to widespread adoption, as a lack of familiarity with the grid’s framework could stall efforts to integrate it into decision-making processes.

### Cultural Biases and Ageism

The grid challenges ingrained cultural biases, particularly the assumption that older adults are inherently frail or cognitively impaired simply due to age. Ageism often leads to overprotective decisions even when the older adult may still have the capacity to make autonomous choices. Overcoming these assumptions is critical for it to be effectively implemented. Shifting the narrative away from blanket protections toward a more person-centered approach, while not insurmountable, is a significant barrier.

In addition, cultural perceptions of safety and independence may vary, as values regarding family involvement versus individualism differ across cultural, ethnic, and socioeconomic groups. For instance, in some cultures, collective decision-making prioritizes family involvement over individualism, which may influence how the balance between safety and independence is approached.

Understanding and addressing these cultural variations is crucial to ensuring the grid remains adaptable and equitable across diverse populations. The grid is not intended as a fixed model but as a flexible framework that can be modified or reimagined to reflect the values, priorities, and lived experiences of those who use it.

### Interdisciplinary Collaboration Challenges

For the grid to function effectively across legal, healthcare, and social services settings requires interdisciplinary collaboration. Differences in professional cultures and priorities could lead to difficulties in aligning the grid’s framework with the expectations and practices of each sector. For example, while court appointed visitors or examiners in guardianship cases may be well-versed in community-based services that support less restrictive alternatives to guardianship, judges and their staff may not be positioned to question whether all avenues outside of guardianship have been exhausted.

### Practical Integration Across Touchpoints

Even if the grid is adopted, consistent application across key decision-making stages remains a challenge. In healthcare settings, for example, hospital discharge planning involves multiple stakeholders determining ongoing care. If the grid is not integrated at this critical juncture, opportunities to ensure decisions reflect the older adult’s preferences, rather than defaulting to safety-first measures, may be lost. Without deliberate use at these touchpoints, the grid risks being overlooked or underutilized, thereby limiting its transformative potential.

## Conclusion and Future Directions

The Safety–Autonomy Grid offers a novel framework for navigating the complex tension between safety and autonomy across multiple levels of decision-making. Whether applied at the individual level—where older adults make decisions about their own care—or at the family or institutional levels, the grid provides a structured tool that helps individuals evaluate their decisions.

From family caregivers and healthcare workers to legal professionals and policymakers, the grid encourages reflection on whether safety and autonomy are being appropriately considered, and whether personal or systemic biases may be skewing decisions in one direction or the other. Through this model, people can better understand the trade-offs they make, evaluate the impact of those choices on an older adult’s well-being, and work toward more balanced, person-centered outcomes.

By visually mapping these priorities, the grid promotes a deeper understanding of the trade-offs involved in caregiving, legal, and policy decisions. At its core, the grid emphasizes that safety and autonomy are not mutually exclusive. Instead, they exist along a continuum where the right balance can—and should—shift depending on the older adult’s specific circumstances.

At the family level, the grid can guide meaningful conversations about care preferences, fostering collaboration and understanding. In institutional contexts, such as healthcare or long-term care, it can inform person-centered care plans that preserve dignity and autonomy while addressing safety concerns. Legal systems may use the grid to evaluate less restrictive alternatives in guardianship cases, safeguarding civil liberties and ensuring balanced protections.

Looking ahead, the Safety–Autonomy Grid should be tested in real-world environments to further refine its applicability. Future directions for the Safety–Autonomy Grid include pilot programs in both legal and healthcare settings to assess its practical impact. Additionally, training programs can incorporate the grid as a tool for professionals across sectors to use in decision-making. Researchers may also explore how the grid can be adapted across cultural contexts, ensuring that it remains relevant for diverse populations.

Ultimately, the Safety–Autonomy Grid transcends its role as a theoretical construct. It is a versatile and pragmatic tool designed to transform decision-making across varied contexts. Whether guiding individuals in their own aging journey, supporting families in care decisions, or shaping institutional policies, the grid fosters thoughtful reflection and balanced priorities, contributing to a future where dignity, autonomy, and safety coexist harmoniously.

## Data Availability

No data were generated or analyzed for this conceptual manuscript.
